# Supplementary material for: Netcombin: An algorithm for constructing optimal phylogenetic network from rooted triplets
Source: PLoS One. 2020 Sep 18;15(9):e0227842. doi: 10.1371/journal.pone.0227842 (PMC7500971; doi:10.1371/journal.pone.0227842)
Supplement: S4 File — (ZIP) [file pone.0227842.s004.zip › SequenceData-Triplet/sequenceData-10-20-30-0-Triplet/length 500, numseq 30, rate 10^-7/6 seq.pdf]

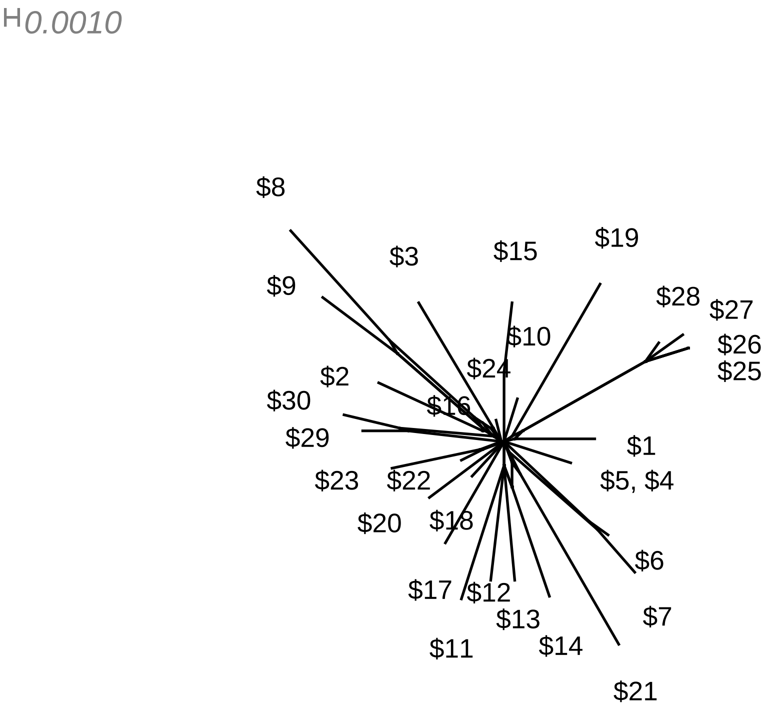
0.0010
$8
$3
$15
$19
$9
$28
$27
$10
$26
$24
$25
$2
$30
$16
$29
$1
$23
$22
$5, $4
$20
$18
$6
$17
$12
$13
$7
$11
$14
$21
